# Deep learning in fracture detection: a narrative review

**DOI:** 10.1080/17453674.2019.1711323

**Published:** 2020-01-13

**Authors:** Pishtiwan H S Kalmet, Sebastian Sanduleanu, Sergey Primakov, Guangyao Wu, Arthur Jochems, Turkey Refaee, Abdalla Ibrahim, Luca v. Hulst, Philippe Lambin, Martijn Poeze

**Affiliations:** a Maastricht University Medical Center+, Department of Trauma Surgery, Maastricht;; b The D-Lab: Decision Support for Precision Medicine, GROW—School for Oncology and Developmental Biology, Maastricht University Medical Center+, Maastricht;; c Nutrim School for Nutrition, Toxicology and Metabolism, Maastricht University, Maastricht, The Netherlands

## Abstract

Artificial intelligence (AI) is a general term that implies the use of a computer to model intelligent behavior with minimal human intervention. AI, particularly deep learning, has recently made substantial strides in perception tasks allowing machines to better represent and interpret complex data. Deep learning is a subset of AI represented by the combination of artificial neuron layers. In the last years, deep learning has gained great momentum. In the field of orthopaedics and traumatology, some studies have been done using deep learning to detect fractures in radiographs. Deep learning studies to detect and classify fractures on computed tomography (CT) scans are even more limited. In this narrative review, we provide a brief overview of deep learning technology: we (1) describe the ways in which deep learning until now has been applied to fracture detection on radiographs and CT examinations; (2) discuss what value deep learning offers to this field; and finally (3) comment on future directions of this technology.

The demands for radiology services, e.g., magnetic resonance imaging (MRI), computed tomography (CT), and radiographs, have increased dramatically in recent years (Kim and Mac­Kinnon 2018). In the United Kingdom, the number of CT examinations increased by 33% between 2013 and 2016 (Faculty of Clinical Radiology, Clinical Radiology UK workforce census 2016 report 2016). In the Netherlands, more than 1.7 million CT examinations were carried out in all hospitals (National Institute for Health and Environment 2016). This demand will increase substantially in the coming years resulting in a considerable strain on the workforce. On the other hand, there is a shortage of radiologists due to a lag in recruitment and the large number of radiologists approaching retirement. Furthermore, analyzing medical images can often be a difficult and time-consuming process. Artificial intelligence (AI) has the potential to address these issues (Kim and Mac­Kinnon 2018).

AI is a general term that implies the use of a computer to model intelligent behavior with minimal human intervention (Hamet and Tremblay 2017). Furthermore, AI, particularly deep learning, has recently made substantial strides in the perception of imaging data allowing machines to better represent and interpret complex data (Hosny et al. 2018).

Deep learning is a subset of AI represented by the combination of artificial neuron layers. Each layer contains a number of units, where every unit is a simplified representation of a neuron cell, inspired by its structure in the human brain (McCulloch and Pitts 1943). Today, deep learning algorithms are able to match and even surpass humans in task-specific applications (Mnih et al. 2015, Moravčík et al. 2017). Deep learning has transformed the field of information technology by unlocking large-scale, data-driven solutions to what once were time-intensive problems.

In the last years, deep learning has gained great momentum (Adams et al. 2019). Recent studies have shown that deep learning has the ability to perform complex interpretation at the level of healthcare specialists (Gulshan et al. 2016, Esteva et al. 2017, Lakhani and Sundaram 2017, Lee et al. 2017, Olczak et al. 2017, Ting et al. 2017, Tang et al. 2018). In the field of orthopaedic traumatology, a number of studies have been done using deep learning in radiographs to detect fractures (Brett et al. 2009, Olczak et al. 2017, Chung et al. 2018, Kim and Mac­Kinnon 2018, Lindsey et al. 2018, Adams et al. 2019, Urakawa et al. 2019). However, studies performing deep learning in fractures on CT scans are scarce (Tomita et al. 2018).

In this narrative review, we provide a brief overview of deep learning technology; (2) describe the ways in which deep learning has been applied to fracture detection on radiographs and CT examinations thus far; (3) discuss what value deep learning offers to this field; and finally (4) comment on future directions of this technology.

## Artificial intelligence technology

**Figure 1.**
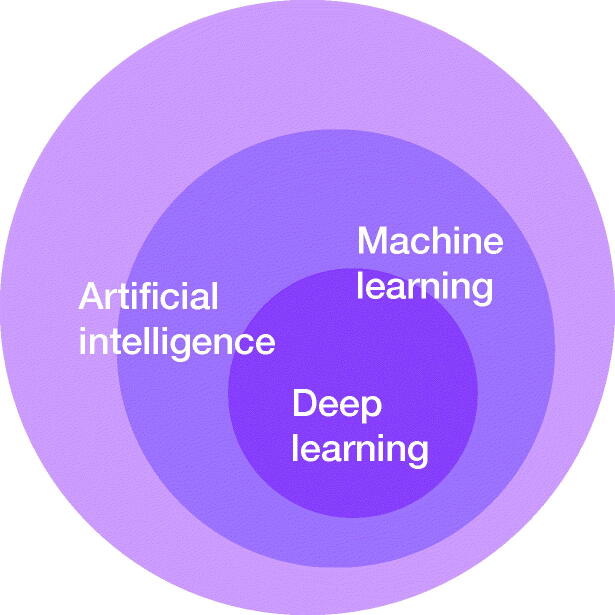
Visualization of Artificial Intelligence sub-family.

Deep Learning (DL) is a family of methods, which is part of a broad Machine-learning field and an even broader Artificial Intelligence field (Figure 1). These algorithms are unified by the idea of learning from data instead of following explicitly specified instructions. This level of abstraction makes Deep Learning algorithms applicable to solve a variety of problems in a number of quantitative fields (LeCun et al. 2015).

Deep Learning has showed outstanding performance for solving semantic image processing tasks. Cireşan et al. (2012) demonstrated that DL can outperform humans by a factor of 2 in traffic sign recognition. Tompson et al. (2014) have shown that DL has significantly outperformed existing state-of-the-art techniques for human pose estimation. Chen et al. (2015) assessed DL potential in autonomous driving application. ImageNet (Russakovsky et al. 2015) demonstrated that DL can be successfully applied to a variety of image-specific tasks and gain state-of the-art performance. After the DL success in the computer vision field, the medical imaging field started to adopt these methods for solving its own problems such as, e.g., medical image classification (Gao et al. 2017, Yang et al. 2018, Tran et al. 2019), medical image segmentation (Cha et al. 2016, Dou et al. 2017, Roth et al. 2018) and noise reduction (Chen et al. 2017, Wolterink et al. 2017). Due to the high abstractness of DL algorithms, there is no need to change methodology when moving from a problem in one field to another field. Moreover, by using this so-called transfer learning approach, DL algorithms are able to benefit from previous successes even if the model was solving a different problem (Yang et al. 2018).

**Figure 2.**
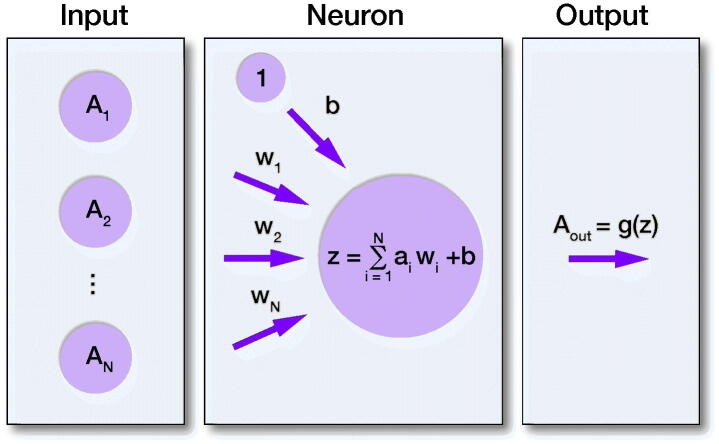
Visualization of artificial neuron model. Where A_1_–A_N_ are the inputs, W_1_–W_N_ are the weights for the input connections to neuron, b is the bias value, z is the output from the neuron.

The essential DL layer is composed of a number of neurons that to a certain extent mimic the activity of a neuron cell (Figure 2). Every neuron in the layer has its own weight w for each input connection and the bias value b, where each weight w represents the strength for the particular connection, and the bias value b allows us to shift the activation function along with the weighted sum of the inputs to the neuron, controlling the value at which the activation function will trigger. In other words, each weight w defines how much influence the corresponding input will have on the neuron output and bias b, allowing the model to better fit the data. In order to create the output for the neuron and introduce non-linearity to the neuron decision, one of the activation functions, g, is applied to the neuron output z.

Expanding this interaction logic for the rest of the neurons, we get the DL layer. The layer where all possible connections between input nodes and output nodes are introduced is called the “Dense layer.” In order to learn more complex features and prevent overfitting, the too close fitting of the model to a limited set of data points in the training dataset, another type of layers was introduced such as the “Convolution layer,” “Pooling layer” and “Dropout layer.” Given the DL model built from such layers and the representative dataset describing the problem we can solve the weights optimization task by using one of the optimization algorithms, e.g., Gradient Descent (GD). GD is used to find a minimum of the cost function by iteratively moving in the direction of steepest descent. It is used due to computational limitations we meet trying to solve the optimization task analytically. The cost function quantifies the error between predicted and the ground truth labels. By calculating the derivative of the error with respect to each neural network weight we obtain the individual gradients, which are subsequently used to update the weights for all corresponding neuron connections. The described procedure represents 1 cycle of the neural network (NN) training process. During the model training process every image from the training dataset contributes to the weights optimization. Thereby, the DL model learns to solve the problem directly from data.

Finding and classification of fractures on radiographs and CT images with high sensitivity and specificity can be assisted or even replaced by the automated DL system with high accuracy. Given a few thousand images we can address several problems with DL. Using such models as VGG16 (Simonyan and Zisisserman 2015), Inception V3 (Szegedy et al. 2015), and Xception (Chollet 2016), we can classify the images, for example to detect whether there is a fracture, or even differentiate between fracture types. Given the bounding box annotations or labels for the regions of interest, we can train such models as ResNet (He et al. 2016), U-net (Ronneberger et al. 2015), Mask-RCNN (He et al. 2017), Faster-RCNN (Ren et al. 2015) for the fracture detection and segmentation problem. The mentioned DL architectures have been widely used in the DL community and have demonstrated their efficiency in solving such tasks (Ruhan et al. 2017, Li et al. 2018, Couteaux et al. 2019, Li et al. 2019, Lian et al. 2019, Zhu et al. 2019).

## Applications of AI in fracture detection

A number of studies have demonstrated the application of deep learning in fracture detection (Brett et al. 2009, Olczak et al. 2017, Chung et al. 2018, Kim and Mac­Kinnon 2018, Lindsey et al. 2018, Tomita et al. 2018, Adams et al. 2019, Urakawa et al. 2019). In a retrospective study by Kim and Mac­Kinnon (2018), they aimed to identify the extent to which transfer learning from deep convolutional neural networks (CNNs), pre-trained on non-medical images, can be used for automated fracture detection on plain wrist radiographs. Authors used the inception V3 CNN (Szegedy et al. 2015), which was originally trained on non-radiological images for the IMageNet Large Visual Recognition Challenge (Russakovsky et al. 2015). They used a training data set of 1,389 radiographs (manually labeled) to re-train the top layer of the inception V3 network for the binary classification problem. They achieved an AUC of 0.95 on the test dataset (139 radiographs). This demonstrated that a CNN model that has been pre-trained on non-medical images can be successfully applied to the problem of fracture detection on plain radiographs. Specificity and sensitivity reached 0.90 and 0.88 respectively. This level of accuracy surpasses previous computational methods for automated fracture analysis such as segmentation, edge detection, feature extraction (such studies reported sensitivities and specificities in the range of 80–85%). Although this study provides proof of concept, a number of limitations remain. A small discrepancy was found between the training accuracy and the validation accuracy at the end of the training process. This was likely to reflect overfitting. There are several strategies that can be used to minimize overfitting. One strategy would be to use automated segmentation of the most appropriate region of interest; the pixels outside of the region of interest would be cropped from the image so that irrelevant features would not influence the training process. Another strategy to minimize overfitting would be the introduction of advanced augmentation techniques. In addition (too small < [1000:10000]) study population size is often a limiting factor in machine learning field. A large sample corresponds to a more accurate reflection of a true population (Lindsey et al. 2018).

A similar study by Chung et al. (2018) aimed to evaluate the ability of deep learning to detect and classify proximal humerus fractures using plain AP shoulder radiographs. Results of the CNN network were compared with the assessment of specialists (general physicians, orthopedic surgeons, and radiologists). Their total dataset consisted of 1,891 plain AP radiographs and they used a pre-trained ResNet-152 model, which was fine-tuned to their proximal humerus fracture datasets. The trained CNN showed high performance in distinguishing normal shoulders from proximal humerus fractures. In addition, promising results were found for classifying fracture type based on plain AP shoulder radiographs. The CNN exhibited superior performance to that of general physicians and general orthopedic surgeons, and similar performance to that of shoulder specialized orthopedic surgeons. This indicates the possibility of automated diagnosis and classification of proximal humerus fractures and other fractures or orthopaedic diseases diagnosed accurately using plain radiographs (Chung et al. 2018).

The retrospective study by Tomita et al. (2018) aimed to evaluate the ability of deep learning to detect osteoporotic vertebral fractures (OVF) on CT scans and developed a machine learning approach, fully powered by a deep neural network framework, to automatically detect OVFs on CT scans. For their OVF detection system, they used a system that consisted 2 major components: (1) a CNN-based feature extraction module; and (2) an RNN module to aggregate the extracted features and make the final diagnosis. For the processing and extraction of features from CT scans they used a deep residual network (ResNet) (He et al. 2016). Their training dataset consisted of 1,168 CT scans; their validation set consisted of 135 CT scans and their test set consisted of 129 CT scans. The performance of their proposed system on an independent test set matched the level performance of practicing radiologists in both accuracy and F1 (mean of precision and recall) score (Tomita et al. 2018). This automatic detection system has the potential to reduce the time and the manual burden on radiologists of OVF screening, as well as reducing false-negative errors arising in asymptomatic early stage vertebral fracture diagnoses (Tomita et al. 2018). A summary of clinical studies involving computer-aided fracture detection is given in the Table.

## Value of deep learning in radiology/orthopedic traumatology

**Figure 3.**
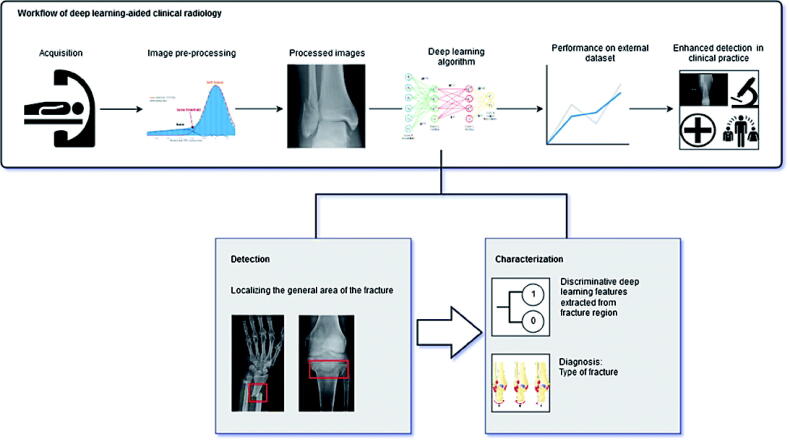
Deep learning aided workflow in fracture detection

As seen from the examples of deep learning in radiology described above, there are potential benefits to the development and integration of deep learning systems in everyday practice, in fracture detection as well as fracture characterization tasks (Figure 3). In general, using deep learning as an adjunct to standard practices within radiology has the potential to improve the speed and accuracy of diagnostic testing while decreasing workforce due to offloading human radiologists from time-intensive tasks. Alongside that, deep learning systems are subject to some of the pitfalls of human-based diagnosis such as inter- and intra-observer variance. Deep learning, applied in academic research settings, can at least match and sometimes exceed human performance in fracture detection and classification on plain radiographs and CT scans.

## Combining deep learning with a radiomics approach

Radiomics is a method that extracts large amount of pre-defined quantitative features from medical images beyond the level of detail accessible to the human eye. Deep learning learns from the entire image, whereas radiomics characterizes only the region of interest of a particular disease. Therefore, it is our opinion that deep learning and radiomics provide complementary imaging biomarkers. Furthermore, as radiomics is more stable in the face of smaller datasets, it is desirable to include these features in models to hedge against the possible overfitting of deep learning networks.

## Future directions

The inclusion of artificial intelligence in decision support systems has been debated for decades (Kahn 1994). As applications of artificial intelligence in radiology/orthopedic traumatology will increase there are several areas of interest that we believe will hold significant value in the future (Brink et al. 2017). There is a consensus that inclusion of AI in radiology/image-based disciplines would enhance diagnostic accuracy (Recht and Bryan 2017). However, there is also a consensus that such tools need to be carefully investigated and interpreted, before integration into clinical decision-support systems.

A future challenge to address will be the radiologists–AI relationship. Jha and Topol (2016) suggested that AI can be used for redundant pattern-recognition tasks, while radiologists focus on cognitively challenging tasks. At large, radiologists would need to have a basic understanding of AI and AI-based tools; however, these tools would not replace radiologists’ work, and their role would not be limited to interpreting AI findings. Rather, AI tools can be used as a complementary tool to confirm/validate radiologists’ doubts and decisions (Liew 2018). Further research regarding radiologists–AI relationship is needed in order to properly integrate these disciplines, including research on how to train radiologists to use AI tools and interpret their results.

AI systems must continue to expand their library of clinical applications. As seen in this review, there are several promising studies that demonstrate how AI can improve our performance on clinical tasks such as fracture detection on radiographs and CT scan, including fracture classifications and treatment decision support.

## Conflict of interest

The authors declare that they have no conflict of interest.

**Table:** Summary of clinical studies involving computer-aided fracture detection

Reference	Region of interest	Modality	Conclusion	Performance (metric)
Olczak et al. 2017	Wrist/Hand/Ankle	Radiographs	This study supports the use of orthopaedic radiographs of artificial intelligence, which can perform at a human level	0.83 (accuracy)
Kim et al. 2018	Wrist	Radiographs	The AUC scores for this test were comparable tostate-of-the-art providing proof of concept for transfer learning from CNNs in fracture detection on plain radiographs	0.95 (AUC) 0.90 (sensitivity) 0.88 (specificity)
Chung et al. 2018	Proximal humerus	Radiographs	The use of artificial intelligence can accurately detect and classify proximal humerus fractures on plain shoulder AP radiographs	Detection: 0.96 (accuracy) 1 (AUC) 0.99 (sensitivity) 0.97 (specificity) Classification: 0.65–0.86 (accuracy) 0.90–0.98 (AUC) 0.88–0.97 (sensitivity) 0.83–0.94 (specificity)
Heimer et al. 2018	Skull	CT	Classification based on the existence of skull fractures on CMIPs with deep learning is feasible	0.97 (AUC) 0.91 (sensitivity) 0.88 (specificity)
Lindsey et al. 2018	Wrist	Radiographs	Deep learning methods are a mechanism by which senior medical specialists can deliver their expertise to generalists on the front lines of medicine, thereby providing substantial improvements to patient care	0.97 (AUC) on Test set1 0.98 (AUC) on Test set2
Tomita et al. 2018	Pelvis	CT	The proposed system will assist and improve OVF diagnosis in clinical settings by pre-screening routine CT examinations and flagging suspicious cases prior to review by radiologists	0.89 (accuracy) 0.91 (F1 score)
Pranata et al. 2019	Calcaneus	CT	The feasibility using deep CNN and SURF for computer-aided classification and detection of the location of calcaneus fractures in CT images	0.98 (accuracy)
Adams et al. 2019	Pelvis	Radiographs	As impressive as recognising fractures is for a DCNN, similar learning can be achieved by top-performing medically naпve humans with less than 1 hour of perceptual training	0.91 (accuracy) 0.98 (AUC)

Abbreviations: CT = computed tomography; AUC = area under curve; CNN = convolutional neural network; AP = plain anteroposterior;

CMIP = curved maximum intensity projections; OVF = Osteoporotic vertebral fractures; SURF = speeded-up robust features;

DCNN = deep convolutional neural networks.
